# Supplementary material for: Rickettsia Infection Benefits Its Whitefly Hosts by Manipulating Their Nutrition and Defense
Source: Insects. 2022 Dec 15;13(12):1161. doi: 10.3390/insects13121161 (PMC9785894; doi:10.3390/insects13121161)
Supplement: Supplementary file 1 [file insects-13-01161-s001.zip › insects-2065933-supplementary.pdf]

Supplement

Table S1. Analysis of variance (ANOVA) on the effects of different concentrations of *Akanthomyces attenuatus* and *Rickettsia* infection on the mortality of whitefly.

Age	Source	*Df*	F	*p*
	Concentration	4	87.47	<0.0001
1st	*Rickettsia*	1	120.33	<0.0001
	Concentration×*Rickettsia*	4	3.86	0.0176
	Concentration	4	312.21	<0.0001
2nd	*Rickettsia*	1	23.77	<0.0001
	Concentration× *Rickettsia*	4	9.06	0.0002
	Concentration	4	126.43	<0.0001
3rd	*Rickettsia*	1	41.95	<0.0001
	Concentration× *Rickettsia*	4	14.19	<0.0001
	Concentration	4	67.24	<0.0001
4th	*Rickettsia*	1	26.77	<0.0001
	Concentration×*Rickettsia*	4	7.92	0.0005

Table S2. Analysis of variance (ANOVA) on the effects of different concentrations of insecticide and *Rickettsia* infection on the mortality of whitefly.

Insecticide	Age	Source	*Df*	F	*p*
Imidacloprid	2nd	Concentration	6	113.77	<0.0001
		Rickettsia	1	3.74	0.063
		Concentration×*Rickettsia*	6	0.83	0.059
	adult	Concentration	6	74.72	<0.0001
		Rickettsia	1	6.05	0.02
		Concentration×*Rickettsia*	6	1.58	0.19
Spirotetramat	2nd	Concentration	6	220.59	<0.0001
		Rickettsia	1	37.97	<0.0001
		Concentration×*Rickettsia*	6	2.98	0.022
	adult	Concentration	6	43.41	<0.0001
		Rickettsia	1	4.06	0.054
		Concentration×*Rickettsia*	6	0.66	0.68
